# Examining Satisfaction With Assisted Living Among Residents With and Without Dementia

**DOI:** 10.1093/geroni/igaa057.1232

**Published:** 2020-12-16

**Authors:** Sarah Holmes, Elizabeth Galik, Barbara Resnick

**Affiliations:** 1 University of Maryland Baltimore Doctoral Program in Gerontology, Baltimore, Maryland, United States; 2 University of Maryland, Baltimore, Maryland, United States; 3 University of Maryland School of Nursing, Baltimore, Maryland, United States

## Abstract

Understanding residents’ satisfaction with assisted living (AL) is essential for creating supportive environments that are focused on residents’ needs and preferences. Nearly half of AL residents experience some level of cognitive impairment, although limited research has examined residents’ satisfaction with AL particularly among those with cognitive impairment. The purpose of this study was to compare satisfaction with AL between residents with and without dementia. Baseline data from the Dissemination and Implementation of Function Focused Care in AL study was used in this analysis. A total of 481 AL residents were included in the sample. Measures included demographic information, Saint Louis University Mental Status Exam (SLUMS), and the Resident Satisfaction Index. Controlling for age, gender, and comorbidities, multivariate analyses of variance was performed to consider the impact of cognitive status on residents’ satisfaction with health care services, physical environment, relationships with staff, and social activities. The majority of participants were female (71%), White (97%), mean age was 89 years old (SD=7.43), and mean SLUMS score was 15.90 (SD=4.74). On average, residents were highly satisfied with AL reporting a mean score of 19.17 (SD=3.15). There were no significant differences in residents’ satisfaction scores (p>.05) between residents with dementia and without dementia across all subdomains of satisfaction: health care services, physical environment, relationships with staff, and social activities. There may have been some bias in results due to social desirability. Further research should explore additional aspects of residents’ satisfaction with staff such as whether or not person-centered care is provided.

